# A Rare Tumour of the Breast: Carcinosarcoma

**DOI:** 10.4021/jocmr2010.03.275w

**Published:** 2010-03-25

**Authors:** Enver Ilhan, Enver Vardar, Guliz Ozkok, Arsenal Sezgin, Safak Sahin, Kenan Teker, Hakan Postaci, Mehmet Yildirim

**Affiliations:** a Department of General Surgery, Izmir Bozyaka Education and Research Hospital, Izmir, Turkey; b Department of Pathology, Izmir Bozyaka Education and Research Hospital, Izmir, Turkey

## Abstract

Carcinosarcoma of the breast, also known as metaplastic carcinoma, is rare with very few cases reported in the literature. A 46-year old female patient presented with a mass in her left breast. Physical examination, ultrasonography and mammography findings were consistent with malignancy. The mass was totally removed. Histopathological examination revealed carcinosarcoma of the breast. Histologic grade of the tumour was III. Ki67 proliferation index was found 40% positive. Tumour cells were positive for p53 (70% positive), c-erb-B2 (5% positive), pancytokeratin and EMA in carcinomatous areas, and vimentin in sarcomatous areas. There was no metastasis in axillary lymph node and distant metastasis. The patient is receiving chemotherapy and is under follow-up in the 54th month. Along with a review of the literature, we present the information regarding the clinical and histological findings and treatment of the patient who was operated due to breast carcinosarcoma.

**Keywords:**

Breast; Carcinoma; Carcinosarcoma

## Introduction

Carcinosarcoma of the breast is a malignant sarcomatoid metaplasia of epithelial carcinoma. This tumor is believed to behave differently from the carcinoma or sarcoma of the breast. There is controversy about its origin. Presentation of the findings about patients diagnosed with breast carcinoma will help demonstrate the characteristics of the disease.

## Case Report

**Figure 1.**
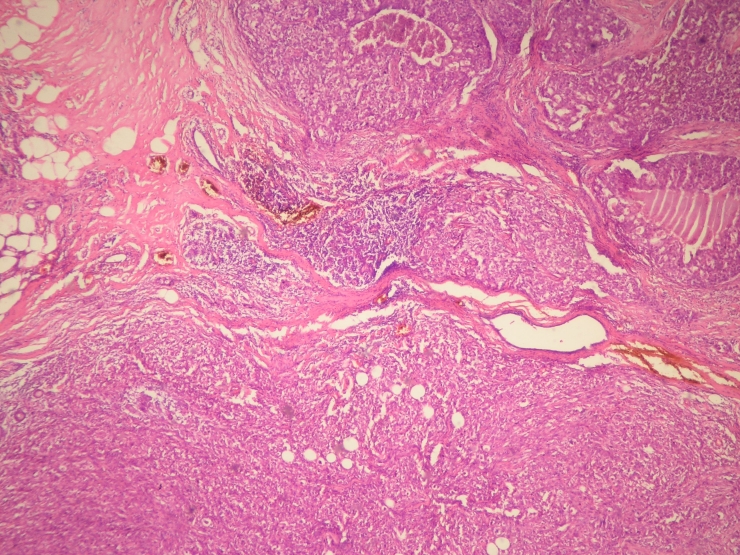
Circular tumor areas containing focally necrosis and infiltration of adipose tissue were seen (HE x 40).

**Figure 2.**
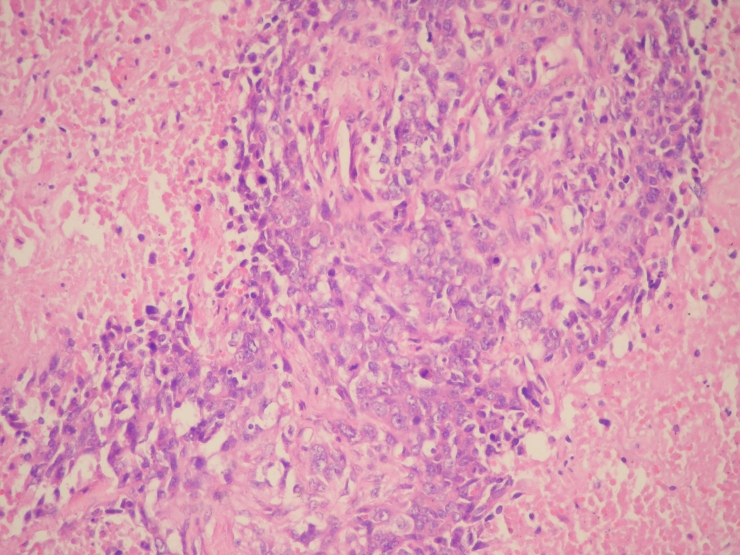
Viable neoplastic cells were seen in between extensive necrosis areas (HE x 200).

**Figure 3.**
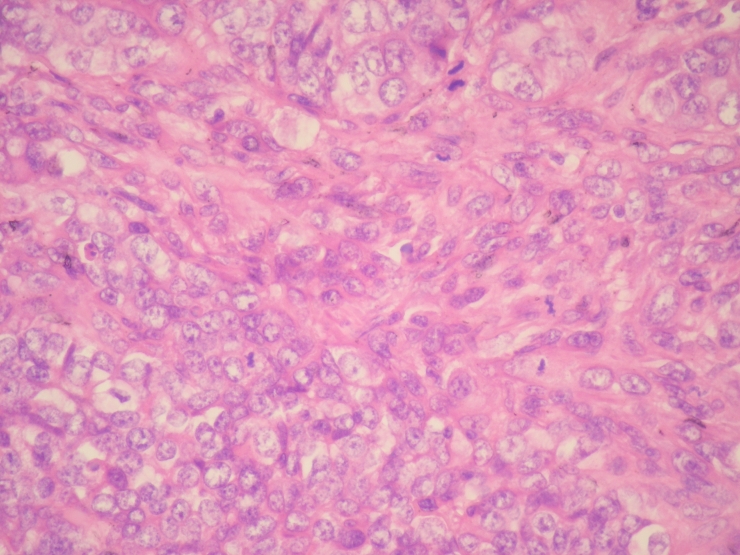
Vesicular chromatin structure and several mitotic figures were seen in tumor cells (HE x 400).

**Figure 4.**
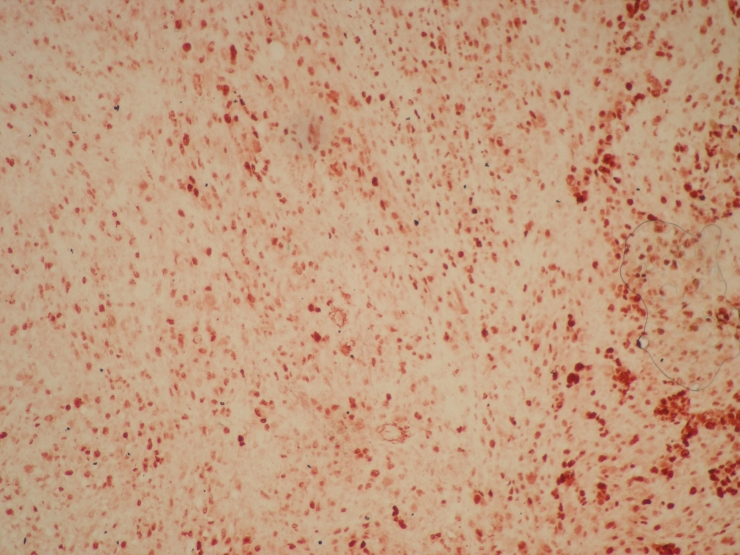
Diffuse and strong nuclear immunohistochemical p53 positivity was seen in tumor cells.

A 46-year old perymenopausal woman (married, having 2 children) presented with a mass present in her right breast which has grown rapidly in 2 months. Physical examination showed a firm mass of 3 cm in greatest dimension with irregular boundaries in the upper inner quadrant of the right breast and no enlarged lymph nodes in the axillary region. Ultrasonography revealed a solid hypoechoic mass of 2.5 cm in greatest dimension displaying irregular boundaries. A radiopaque lesion with irregular boundaries was monitored in mammography. The mass was totally excised under local anesthesia. In gross description the mass measured 2.6 × 2.5 × 2.5 cm and revealed disorganization presenting areas with epithelial and mesenchymal characteristics on histopathological examination. Extensive necrosis was also observed. No lymphovascular thrombi was detected. Epithelial areas with a ductal nature were determined to be of histologic grade III with a total score of 9 (according to the Nottingham modification of Bloom-Richardson grading) consisting of 3 for tubule formation, 3 for nuclear pleomorphism, and 3 for mitotic rate. Ki67 proliferation index was 40% positive. Tumor cells were positive for p53 (70% positive), c-erb-B2 (5% positive),pan-cytokeratin and EMA in carcinomatous areas, and vimentin in sarcomatous areas. All tumor cells were negative for estrogen, progesterone, desmin and SMA (Fig. 1, 2, 3, 4). No metastasis was found in systemic radiological investigations. Right modified radical mastectomy was performed. No residual tumor was found in the excision area in the mastectomy specimen. Histopathological examination of the 26 lymph nodes dissected from the axillary material was consistent with 'reactive hyperplasia'. Having received 6 courses chemotherapy, the case is now in her 54th month and under follow-up with no disease.

## Discussion

Carcinosarcoma of the breast is a malignant sarcomatoid metaplasia of epithelial carcinoma and is rare. It accounts for 0.08 - 0.2% of all malignant breast lesions [1-3]. Carcinosarcoma is a general term describing biphasic lesions simultaneously containing malignant epithelial and malignant mesenchymal tissue components. It has been reported that axillary lymph node of this tumor is less frequently involved and it behaves differently from the carcinoma or sarcoma of the breast with a worse prognosis than classical breast carcinoma [2, 4].

The origin of breast carcinosarcoma is controversial. Myoepithelial cells are believed to originate from a single stem cell like spindle-cells. They have also been reported to develop from existing cystosarcoma phyllodes, fibroadenoma and cystic backgrounds [5-8].

The epithelial component in a tumor may be composed of undifferentiated carcinoma, adenocarcinoma, in situ carcinoma, infiltrative ductal carcinoma or squamous carcinoma. Mesenchymal components may contain different elements ranging from undifferentiated mesenchymal areas to fibroblastic, chondroblastic or osteoblastic areas [9-12].

Clinical findings often reveal swelling in the breast or a palpable mass. Patients usually present with a large mass. Rarely, nipple discharge, nipple retraction or skin ulceration may also be present [9].

Hormone receptors and c-erb-B2 are usually negative [4, 10]. They spread via the blood and lymphatic circulation. The most common distant metastasis areas are lungs [13]. Neoplastic cells disposed to local recurrence as they are prevail in the perivascular tissue [7]. Prevention of local recurrence is of particular importance and radiotherapy is significant in preventing recurrence.

Its diagnosis and treatment involve some difficulties. A multidisciplinary approach is required for treatment. Modified radical mastectomy is preferred in surgical treatment. Anthracycline/taxane-based chemotherapy is recommended for chemotherapy [14]. It is an aggressive type of tumor. However, no significant difference was found when it was compared to high-grade receptor-negative infiltrative carcinomas [4].

Carcinosarcoma of the breast is an aggressive type of breast cancer with worse prognosis than classical breast carcinomas. Tumor size, differentiation rate, high histologic grade, atypia and active pleomorphic spindle cells play a role in prognosis [15].

Recently, Hennessy et al. reported on 100 patients with biphasic metaplastic sarcomatoid carcinoma and 98 patients with carcinosarcoma identified through the SEER database. The authors identified 5-year overall survival (OS) at stage I, II, II and IV as 0.73, 0.59, 0.44, and 0.00, respectively. Our case is recurrence- and metastasis-free in the 54th month under follow-up [4].

Carcinosarcoma of the breast is rare and there are a few numbers of published cases. In order to gain insight into the similar and different characterizing aspects of breast cancer, diagnosed cases should be reported with a literature review and this rare entity should be kept in mind.
